# Urothelial MaxiK-activity regulates mucosal and detrusor metabolism

**DOI:** 10.1371/journal.pone.0189387

**Published:** 2017-12-27

**Authors:** Yi Wang, Gary G. Deng, Kelvin P. Davies

**Affiliations:** 1 Department of Urology, Albert Einstein College of Medicine, Bronx, New York, United States of America; 2 Lilly Research Laboratories, Eli Lilly and Company, Indianapolis, Indiana, United States of America; 3 Department of Physiology and Biophysics, Albert Einstein College of Medicine, Bronx, New York, United States of America; University of Oklahoma Health Sciences Center, UNITED STATES

## Abstract

There is increasing evidence for a role of MaxiK potassium channel-activity in regulating the metabolism and intracellular signaling of non-contractile bladder mucosal tissues. At present however no studies have determined the impact of urothelial MaxiK-activity on overall bladder metabolism. To address this we have investigated the effect of bladder lumen instillation of the MaxiK inhibitor, iberiotoxin (IBTX), on mucosal and detrusor metabolism using metabolomics. Since IBTX does not cross plasma membranes, when instilled into the bladder lumen it would only effect urothelially expressed MaxiK-activity. Surprisingly IBTX treatment caused more effect on the metabolome of the detrusor than mucosa (the levels of 17% of detected detrusor metabolites were changed in comparison to 6% of metabolites in mucosal tissue following IBTX treatment). In mucosal tissues, the major effects can be linked to mitochondrial-associated metabolism whereas in detrusor there were additional changes in energy generating pathways (such as glycolysis and the TCA cycle). In the detrusor, changes in metabolism are potentially a result of IBTX effecting MaxiK-linked signaling pathways between the mucosa and detrusor, secondary to changes in physiological activity or a combination of both. Overall we demonstrate that urothelial MaxiK-activity plays a significant role in determining mitochondrially-associated metabolism in mucosal tissues, which effects the metabolism of detrusor tissue. Our work adds further evidence that the urothelium plays a major role in determining overall bladder physiology. Since decreased MaxiK-activity is associated with several bladder pathophysiology’s, the changes in mucosal metabolism reported here may represent novel downstream targets for therapeutic interventions.

## Introduction

The correct regulation of bladder tone is essential for its normal function; perturbations in the regulation of detrusor smooth muscle contractility are associated with changes in bladder voiding, resulting in such conditions as over- or under- active bladder. In this regard, the importance of MaxiK (a Ca^2+^- and voltage-gated potassium channel (a.k.a. BK, KCNMA1, KCa1.1, encoded by the *slo* gene) in normal bladder function has been shown unequivocally through the use of *slo*^-/-^ knock-out mice [1,2]. In these mice, absence of MaxiK results in enhanced myogenic and nerve-mediated detrusor contractility and increased voiding frequency. In addition protein levels of MaxiK are down-regulated in bladder from rabbits with partial urethral obstruction (PUO), a model of overactive bladder [3]. Our own studies in the PUO animal model have demonstrated that gene transfer of pVAX-Slo (a plasmid construct expressing the *slo* gene encoding the MaxiK pore-forming subunit) into the bladder reduced over-activity [4].

Alterations in MaxiK-activity are at present believed to impact bladder physiology primarily through regulation of detrusor smooth muscle contractility. Because of this concept, until recently most studies on the function of MaxiK in bladder physiology have focused on its role in detrusor and rarely differentiate between its presence or potential different role in other tissue compartments of the bladder. However, it is increasingly recognized that MaxiK-activity is present in the non-contractile tissues of the bladder (the mucosa: consisting of the urothelium, lamina propria and microvasculature) where its function is not well-defined [5,6]. Recent studies have suggested that MaxiK plays a role in regulating metabolism and intercellular signaling which may be more relevant to its function in the non-contractile tissues of the bladder [7–9]. In patients with overactive bladder (OAB) it has been reported that there is an association between polyamine metabolism and MaxiK-activity in the urothelium [6,10]. In addition, the need to study mucosal and detrusor metabolism as separate compartments of the bladder is supported through the use of metabolomics; our recently published paper demonstrated significantly different changes in the mucosa and detrusor caused by hyperglycemia [11].

As yet no studies have been published mapping the impact of MaxiK-activity on global cellular metabolism in tissues of the bladder. To address this we have used metabolomics to determine the role of urothelial MaxiK-activity in regulating global mucosal metabolism *in vivo* by using a specific inhibitor of MaxiK activity (iberiotoxin, IBTX) instilled into the lumen of the bladder. Iberiotoxin is a hydrophilic compound, which has been shown by numerous studies not to penetrate the intact cellular membrane and will therefore only effect MaxiK-activity on the luminal surface of the bladder (the urothelium). However, given the emerging role of the mucosa in regulating overall bladder function, we also determined if changes in mucosal metabolism would then impact detrusor metabolism.

## Materials and methods

### Animals and tissues

All experimental protocols were approved by the Institutional Animal Care and Use Committee of the Albert Einstein College of Medicine. Sixteen F344 rats were separated into two groups that received either IBTX (1mg/ml) or vehicle (phosphate buffered saline, PBS) for one hour via bladder lumen instillation. Urodynamic analysis through continuous flow cystometry (as described below) was performed for one hour following IBTX instillation. Upon completion of cystometry animals were sacrificed and the bladders were removed and immediately placed into cold phosphate buffered saline (137mM NaCl, 8mM Na_2_HPO_4_, 2.7mM KCl, 1.47mM KH_2_PO_4_, pH7.4) to separate the detrusor and urothelium. Tissue was then transferred into tubes and stored in -80°C degree prior to shipping for metabolomic profiling by Metabolon Corp. (as previously described [11]).

### Urodynamic analysis

Procedures for urodynamic analysis have been previously described by our laboratory [12]. Urodynamics was through continuous flow cystometry in conscious rats through infusion of room temperature saline at a rate of 10mL/h to elicit repetitive micturition. The following urodynamic parameters were quantified: (a) Bcap = bladder capacity (volume of infused saline at micturition); (b) MV = micturition volume (volume of urine discharged during micturition); (c) RV = residual volume (volume of infused saline minus the micturition volume); (d) BP = basal pressure (lowest average bladder pressure recorded during cystometry; (e) TP = threshold pressure (intravesical pressure at which voiding is triggered); (f) MP = micturition pressure (peak intravesical pressure during voiding); (g) IMP = intermicturition pressure (average pressure between micturitions); (h) SA = spontaneous activity (an approximate index of spontaneous detrusor contractions between voiding and; (i) Bcom = bladder compliance (calculated by subtracting the basal pressure from the IMP).

### Statistical analysis

The value for each cystometric measurement was determined for each animal after IBTX (or PBS (vehicle control)) instillation and the mean calculated. Differences were evaluated through T-test with statistical significance defined as *p*<0.05.

The metabolomics data set was first normalized and mean-centered. Then, the data were subjected to unsupervised statistical analysis, principal component analysis (PCA) to classify the samples (control and IBTX-treated). Subsequently, all the data was subjected to ANOVA contrast analysis for specific comparison between IBTX-treated groups and control groups. Analysis by two-way ANOVA with repeated measures identified metabolites exhibiting significant interaction and main effects for the experimental parameter of IBTX treatment. The *p*-value is the probability that the test statistic is at least as extreme as observed in this experiment given that the null hypothesis is true. The significance levels were adjusted for multiple hypothesis testing at a false discovery rate (FDR) of 5%, which can be estimated using the q-value.

## Results

### Cystometry demonstrates the effect of one hour of instillation of IBTX on urodynamic parameters is limited

In Table 1 we compare cystometric parameters measured after luminal installation of IBTX or the vehicle control. The only urodynamic parameter that was significantly (*p*<0.05) affected by IBTX treatment was micturition pressure (MP) which was reduced. Although it might be expected that in a non-compliant bladder the MP would be elevated this observation may represent a specific effect of MaxiK rather than this being a model of a non-compliant bladder.

**Table 1:** The effect of iberiotoxin on cystometric parameters.

	Bcap (ml)	MV (ml)	RV (ml)	BP (cm H_2_O)	TP (cm H_2_O)	MP (cm H_2_O)	IMP (cm H_2_O)	SA (per hour)	Bcom (ml/cm H_2_O)
**Following IBTX treatment**	0.63±0.08	0.54±0.07	0.09±0.03	9.85±1.97	15.08±1.92	38.96±4.62*	13.06±2.03	3.21±0.24	0.24±0.09
**Following PBS treatment**	0.76±0.06	0.80±0.10	-0.04±0.15	8.63±0.95	19.42±1.41	58.61±1.80	14.03±1.54	5.40±1.31	0.07±0.01

Note: The mean value of each cystometric parameter (± standard deviation) is shown after luminal instillation of IBTX. The following abbreviations were used (a) Bcap = bladder capacity (volume of infused saline at micturition); (b) MV = micturition volume (volume of urine discharged during micturition); (c) RV = residual volume (volume of infused saline minus the micturition volume); (d) BP = basal pressure (lowest average bladder pressure recorded during cystometry; (e) TP = threshold pressure (intravesical pressure at which voiding is triggered); (f) MP = micturition pressure (peak intravesical pressure during voiding); (g) IMP = intermicturition pressure (average pressure between micturitions); (h) SA = spontaneous activity (an approximate index of spontaneous detrusor contractions between voiding and; (i) Bcom = bladder compliance (calculated by subtracting the basal pressure from the IMP). The only variable significantly affected by IBTX treatment was MP (* = P<0.05).

### Metabolomics demonstrates inhibition of urothelial MaxiK activity effects the metabolic profile of mucosa and detrusor differently

**Fig 1:**
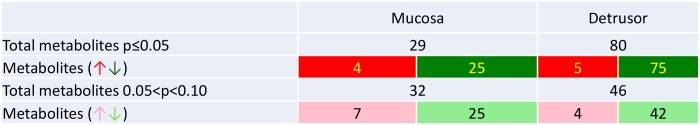
Number of metabolites with significantly changed levels after iberiotoxin treatment. Note: the following key is used for throughout the Figs in the paper: Dark Green Background: Indicates significant (p≤0.05) down-regulation of metabolite following IBTX treatment; Light Green Background: Indicates a trend/narrowly missed statistical significance (0.05<p<0.10) for down-regulation of metabolite following IBTX treatment; Dark Red Background: Indicates significant (p≤0.05) up-regulation of metabolite following IBTX treatment; Light Red Background: Indicates a trend/narrowly missed statistical significance (0.05<p<0.10) for up-regulation of metabolite following IBTX treatment. Grey Background: Indicates a detected metabolite which was unchanged following IBTX treatment. White Background: Indicates an undetected metabolite which is part of the metabolic pathway.

**Fig 2:**
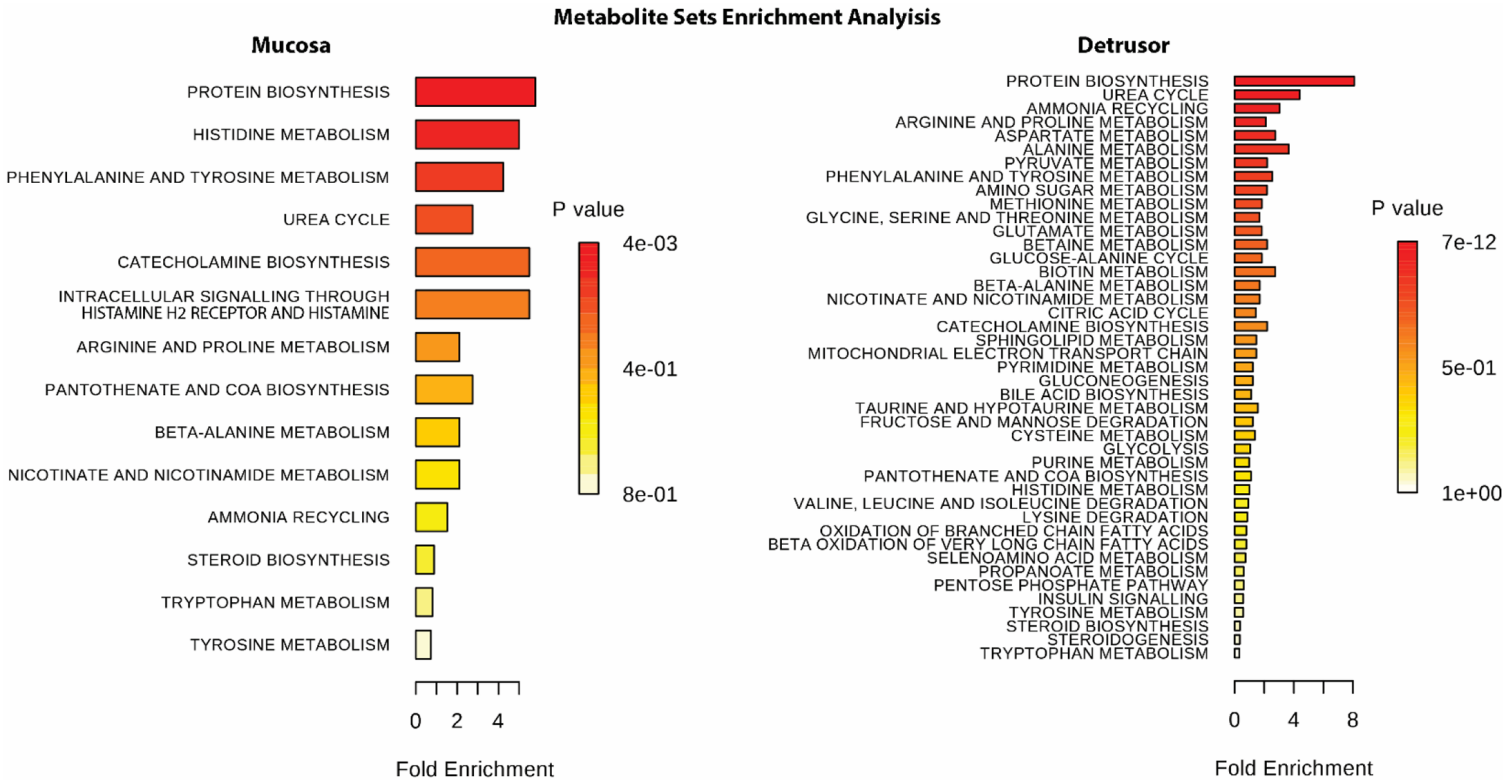
An overview of the involvement of the metabolites sorted by molecular processes in the mucosa and detrusor that are changed in response to IBTX treatment is depicted through an enrichment analysis (using MetaboAnalyst 3.0)[13].

In total, 522 and 484 identifiable metabolites were detected at significant levels in the mucosal and detrusor layers, respectively. As shown in Fig 1, following IBTX treatment the percentage of significant change in the levels of detectable metabolites 6%, *p*≤0.05 (11% *p*≤0.1) in the mucosa and 16%, *p*≤0.05 (26% *p*≤0.1) in the detrusor. A complete list of the detected metabolites and their change following IBTX treatment is presented as supplemental data (S1 Fig). In Fig 2 an overview of the involvement of the metabolites sorted by molecular processes in the mucosa and detrusor that are changed in response to IBTX treatment is depicted through an enrichment analysis (using MetaboAnalyst 3.0) [13]. In the following sections we provide a more detailed analysis and interpretation of these changes.

### Evidence of an effect of urothelial MaxiK inhibition on mucosal and detrusor mitochondrial metabolism

The data presented below demonstrate a role for urothelial MaxiK-activity in regulating mitochondrial metabolism in both mucosal and detrusor tissue. In the mucosa the primary effect appears to be on mitochondrial metabolism, whereas in the detrusor additional pathways are affected.

**Fig 3:**
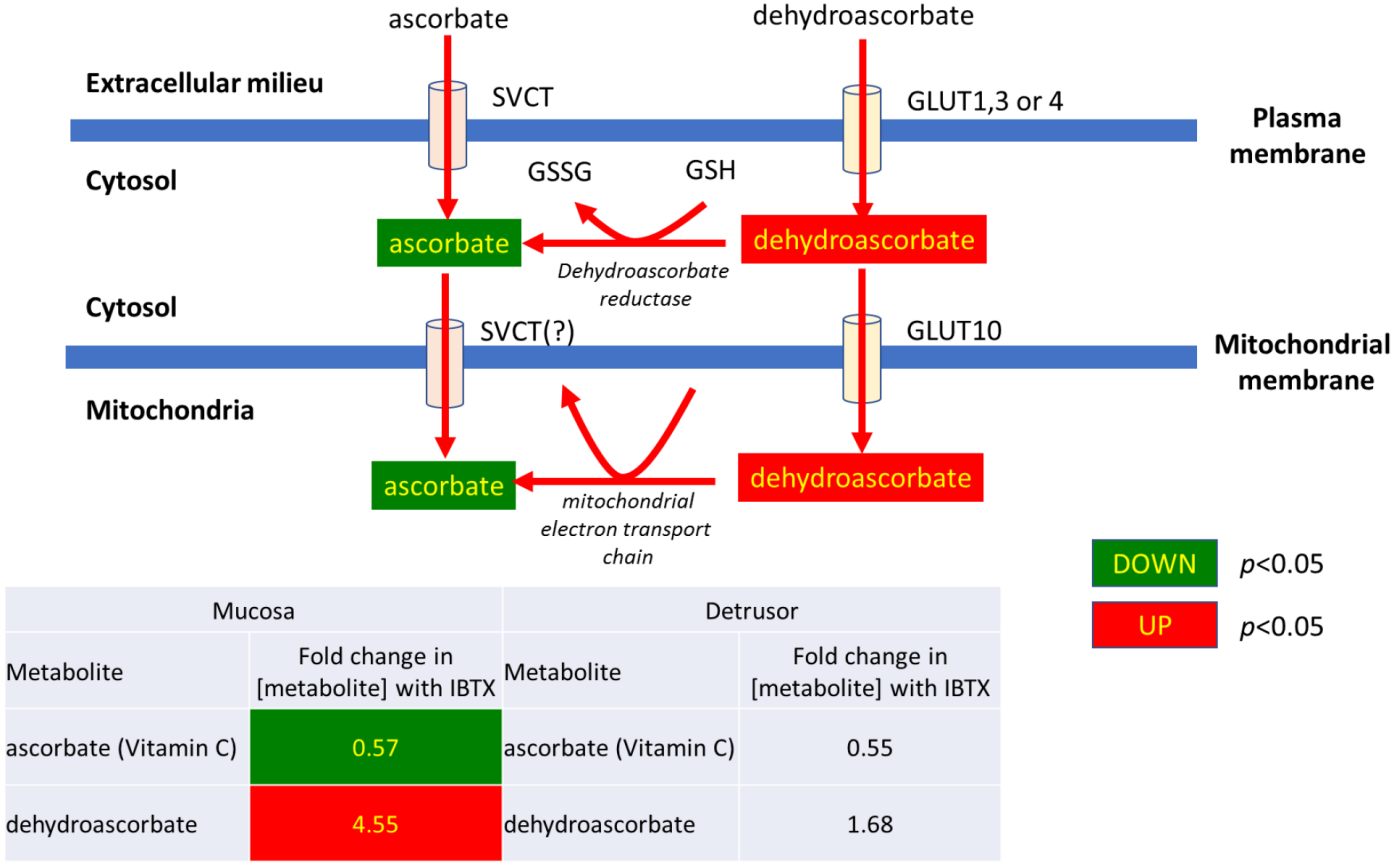
Iberiotoxin treatment causes a significant decrease in the ascorbate/dehydroscorbate ratio in mucosal tissue (and a similar trend was observed in the detrusor).

**1)** As shown in Fig 3, in mucosal tissue IBTX treatment causes a significant decrease in the ascorbate/dehydroscorbate ratio (and a similar trend was observed in the detrusor). Dehydroascorbate is an oxidized form of ascorbic acid, which is transported by GLUT10 into mitochondria, where it is reduced to ascorbate. The changes in metabolite expression suggests that either mitochondrial import, or enzymatic reduction of dehydroascorbate (which would rely on the generation of the mitochondrially derived cofactor, reduced-glutathione (GSH)), are negatively impacted by IBTX.

**Fig 4:**
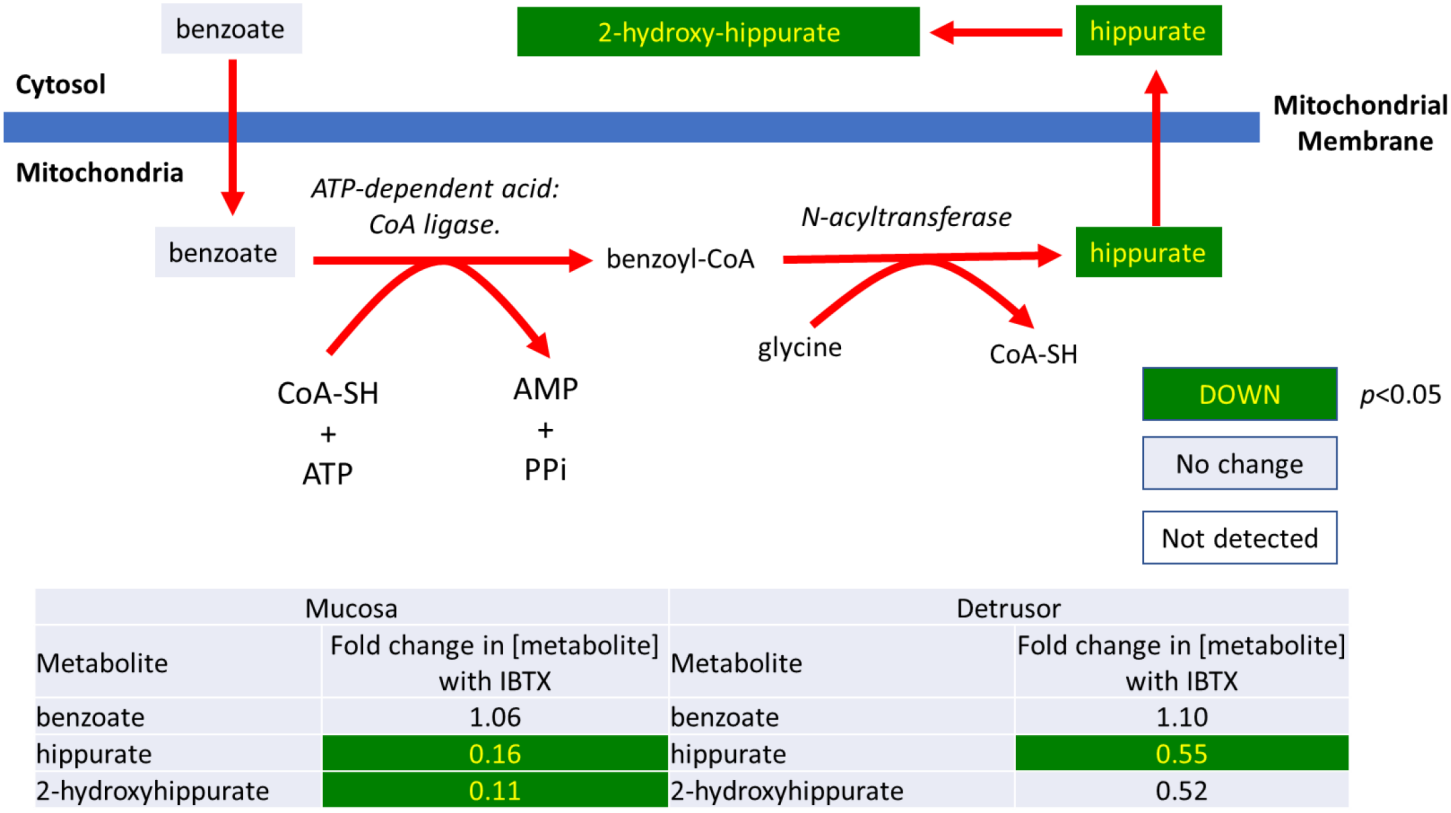
Iberiotoxin treatment causes a significant decrease in the hippurate/benzoate ratio in mucosal and detrusor tissue.

**Fig 5:**
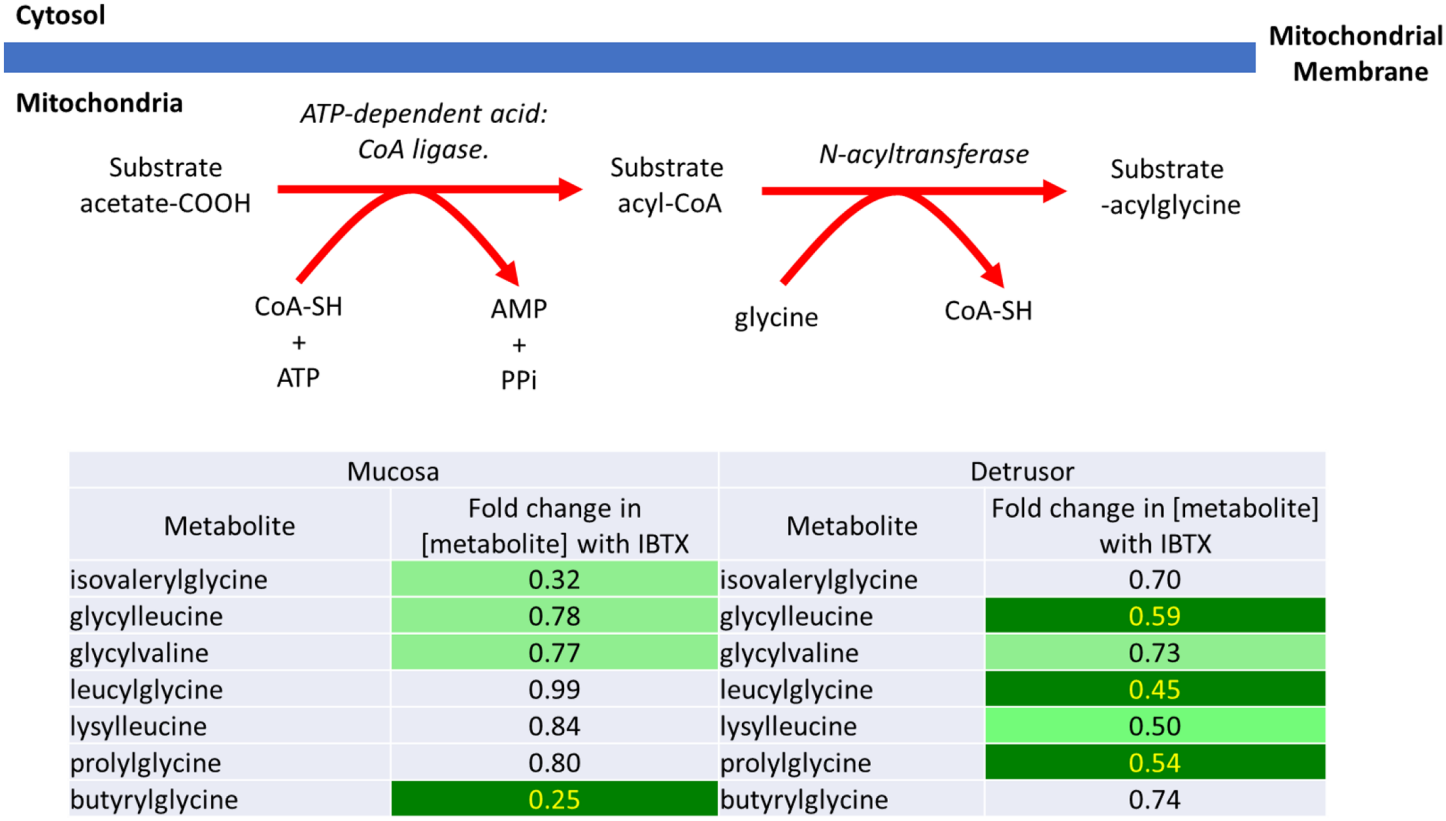
Iberiotoxin treatment causes a significant decrease in the products of glycine N-acyltransferase activity in mucosal and detrusor tissue.

**2)** As shown in Figs 4 and 5, several products of the mitochondrial enzyme, glycine N-acyltransferase, are down regulated after IBTX treatment. For example, as shown in Fig 4, benzoate levels are not effect by IBTX treatment in either urothelium or detrusor, but its downstream metabolites, hippurate and 2-hydroxyhippurate are significantly lower in both tissues [14]. Benzoate is metabolized in the mitochondria to hippurate by the action of an ATP-dependent acid: CoA ligase to produce benzoyl-CoA, which is subsequently converted to hippurate by glycine N-acyltransferase, and then exits the mitochondria. Hippurate can then be further metabolized to 2-hydroxyhippurate. Lowered hippurate levels could reflect either reduced benzoate uptake by the mitochondria, or reduced activity of mitochondrially located N-acyltransferase enzymes, which might occur as a result of reduced levels of co-factors associated with enzymes (Coenzyme-A (CoA-SH) or ATP), which are generated in the mitochondria.

Other products generated through the activity of the mitochondrially located glycine N-acyltransferase activity are also down-regulated by IBTX treatment in both the mucosa and detrusor as shown in Fig 5 [15]. Although the levels of the parent metabolites were not identified in the metabolomic screen, reduced levels of the mitochondrially generated co-factors of N-acyltransferase (CoA-SH or ATP) could be a potential cause for the lower levels of these glycylated-products.

**Fig 6:**
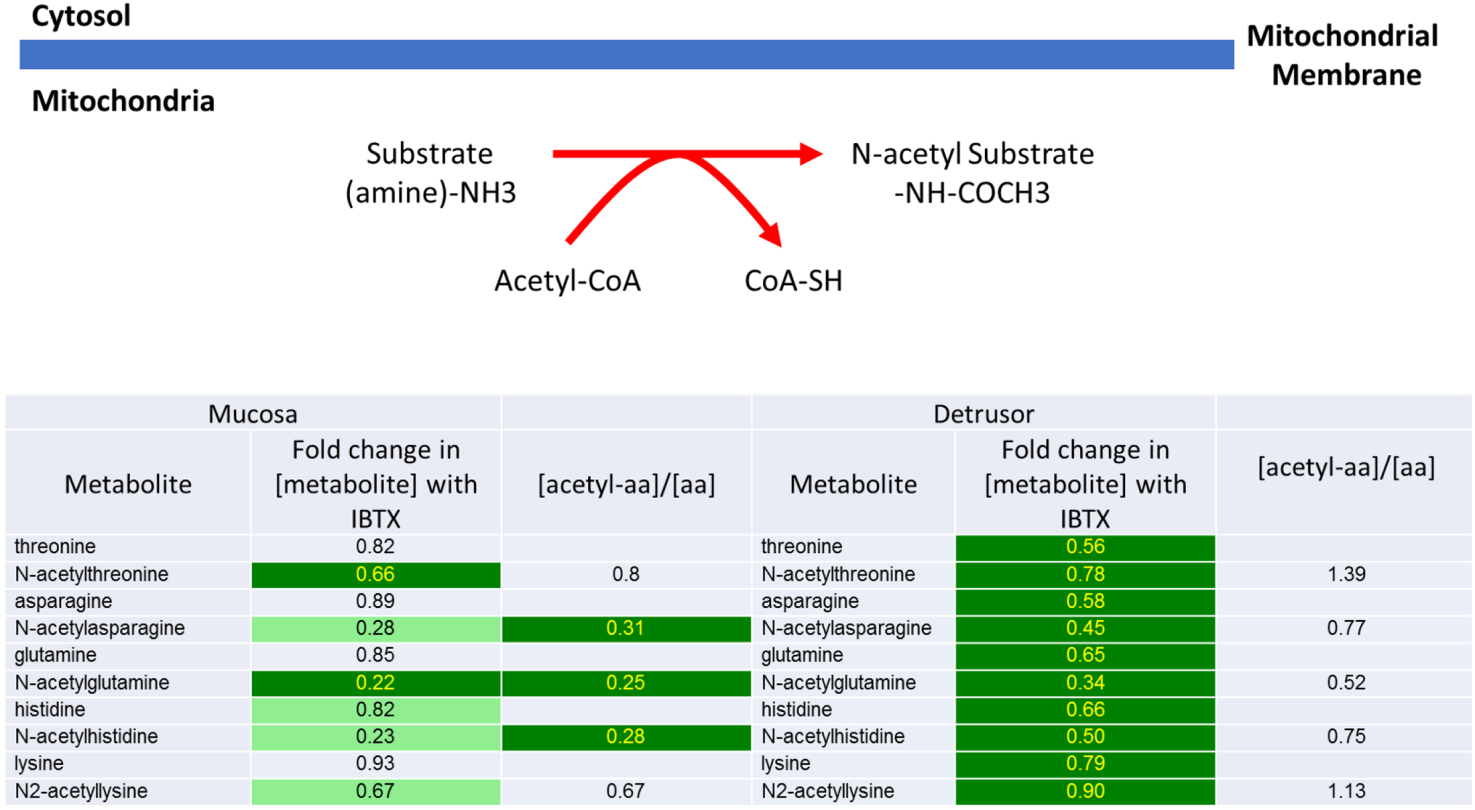
Iberiotoxin treatment causes a significant decrease in the N-acetylated products in mucosal and detrusor tissue.

**Fig 7:**
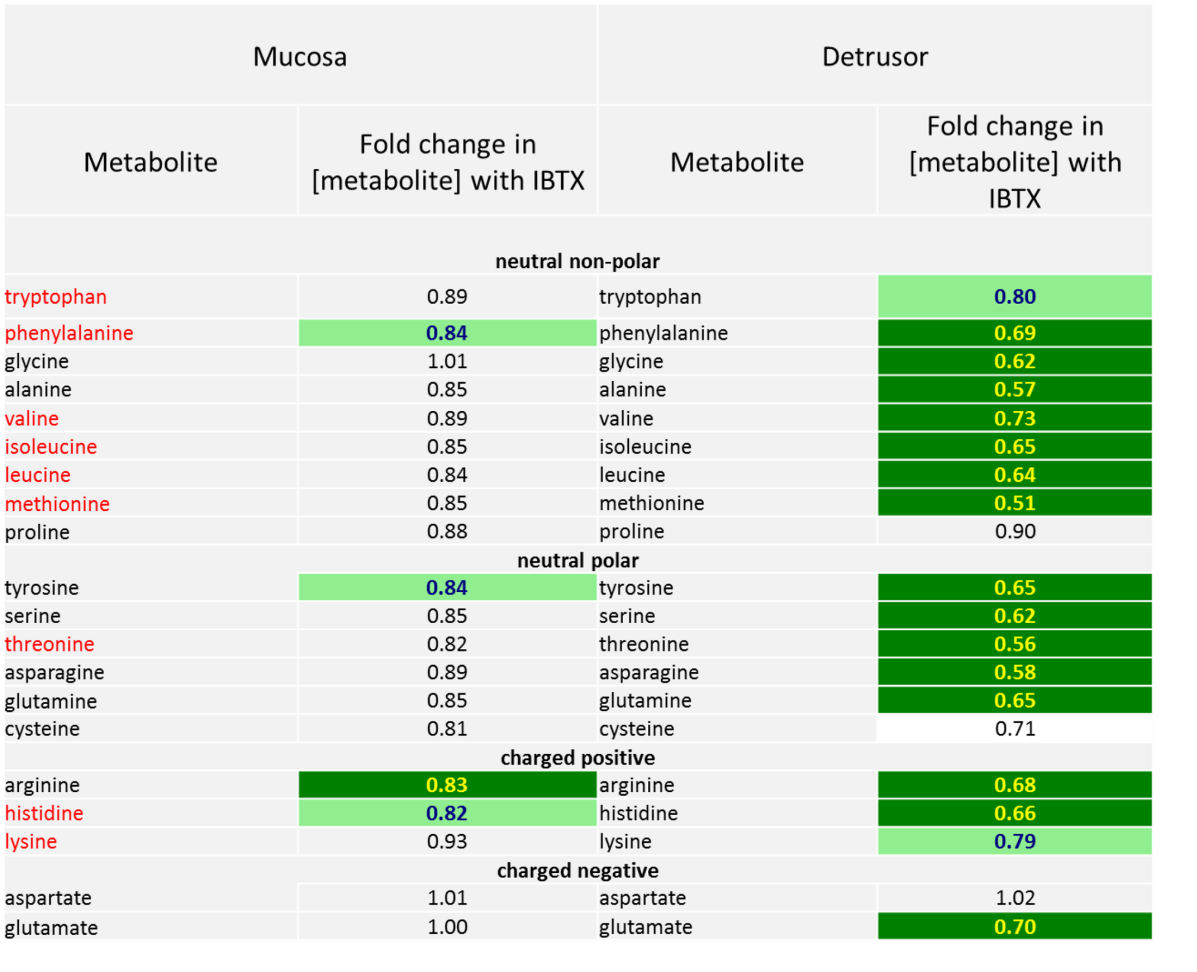
Comparative changes in levels of amino acids in the mucosa and detrusor following iberiotoxin treatment. The amino acids are grouped as neutral non-polar, neutral polar, charged positive and charged negative. Essential amino acids are shown in red.

**3)** As shown in Fig 6 there is a reduction in N-acetylated products of several amino acids (aa) in detrusor and mucosa. In the detrusor, IBTX treatment causes lowered levels of nearly all aa (see Figs 6 and 7) with a concomitant decrease with their N-acetylated metabolites (acetyl-aa). In contrast, in the mucosa the levels of only a few parent aa are significantly affected by IBTX treatment, but there are decreased levels of their N-acetylated products, as illustrated by a decrease in the ratio of acetyl-aa/aa. The N-acetylated metabolites are generated either through direct acetylation of amino acids in the mitochondria, or are the products of protein catabolism in the mitochondria. In the mucosa, the lower ratio of acetyl-aa/aa after IBTX treatment compared to that of detrusor suggests that the amino acids present in mucosa are not being efficiently N-acetylated. In the detrusor, lower levels of nearly all amino acids with a proportionately lower level of their N-acetylated products suggests lowered protein catabolism.

**Fig 8:**
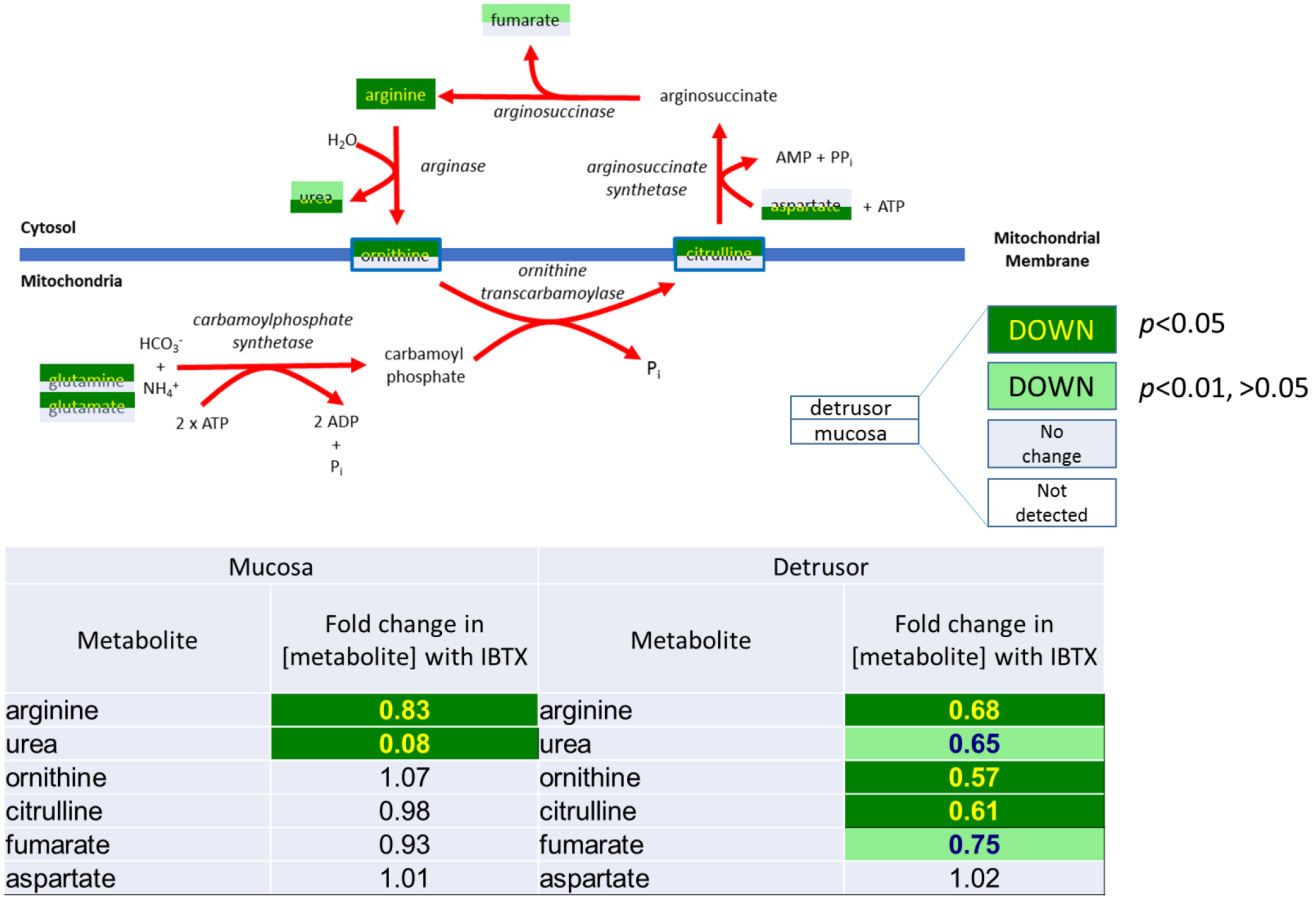
Iberiotoxin treatment causes a significant decrease in the urea cycle products in mucosal and detrusor tissue.

**4)** As shown in Fig 8 IBTX causes reduced levels of urea and arginine in the urothelium, and in the detrusor reduces the levels of all detected intermediary metabolites of the urea cycle. The urea cycle has a critical step that occurs in the mitochondria catalyzed by ornithine transcarbomoylase.

**Fig 9:**
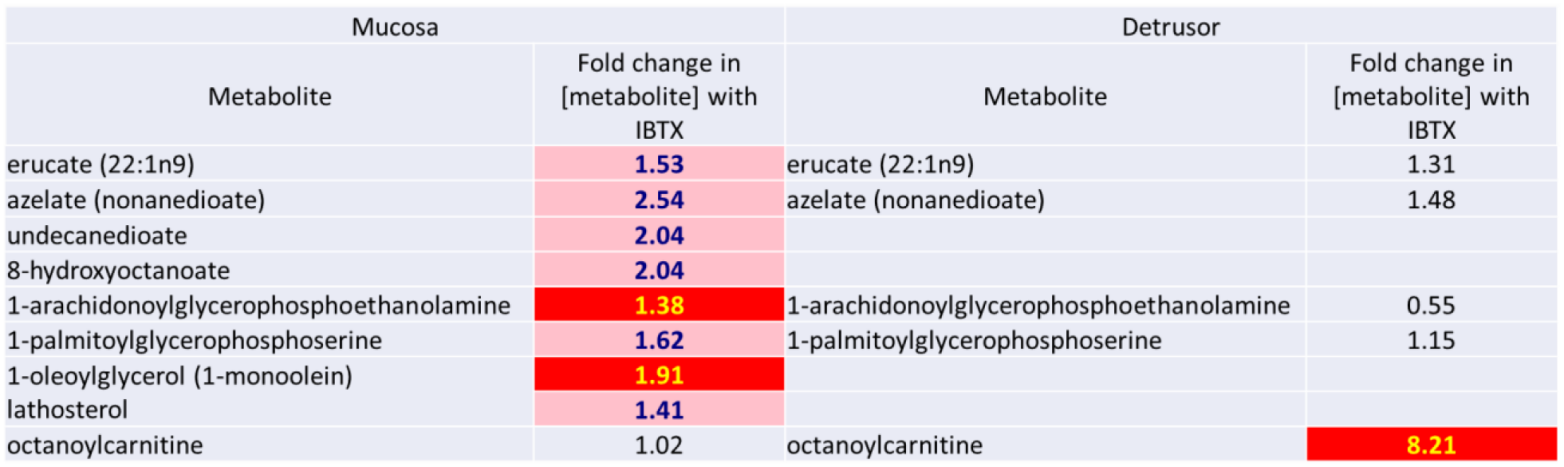
Comparative changes in levels of several intermediary metabolites of lipid metabolism in the mucosa and detrusor following iberiotoxin treatment.KDavies-47.

**5)** As shown in Fig 9 inhibition of MaxiK activity by IBTX causes a significant increase in the levels of several intermediary metabolites of lipid metabolism in the mucosa. Several of these intermediates are fatty acids (such as erucate, azelate, nonanedioate, unndecanedioate and 8-hydroxyoctanoate) that are shortened by cytochrome P450 mediated ω-oxidation in peroxisomes, with the products of these reactions subsequently transported to the mitochondria for complete β-oxidation [16]. Overall fewer intermediary metabolites of lipid peroxidation were detected in the detrusor, and of those that were detected, there was only significantly increased levels of octanoylcarnitine following IBTX treatment (which was not changed in mucosal tissues). At least in rat liver, the enzyme responsible for the synthesis of octanoylcarnitine (carnitine octanoyltransferase) is present only in peroxisomes but not present in mitochondria [17]. These observations suggest that IBTX inhibition of urothelial MaxiK may activate signaling pathways which specifically affects peroxisomal mediated lipid metabolism in the detrusor.

### Histidine metabolism and sulfonation is affected both in the urothelium and detrusor

**Fig 10:**
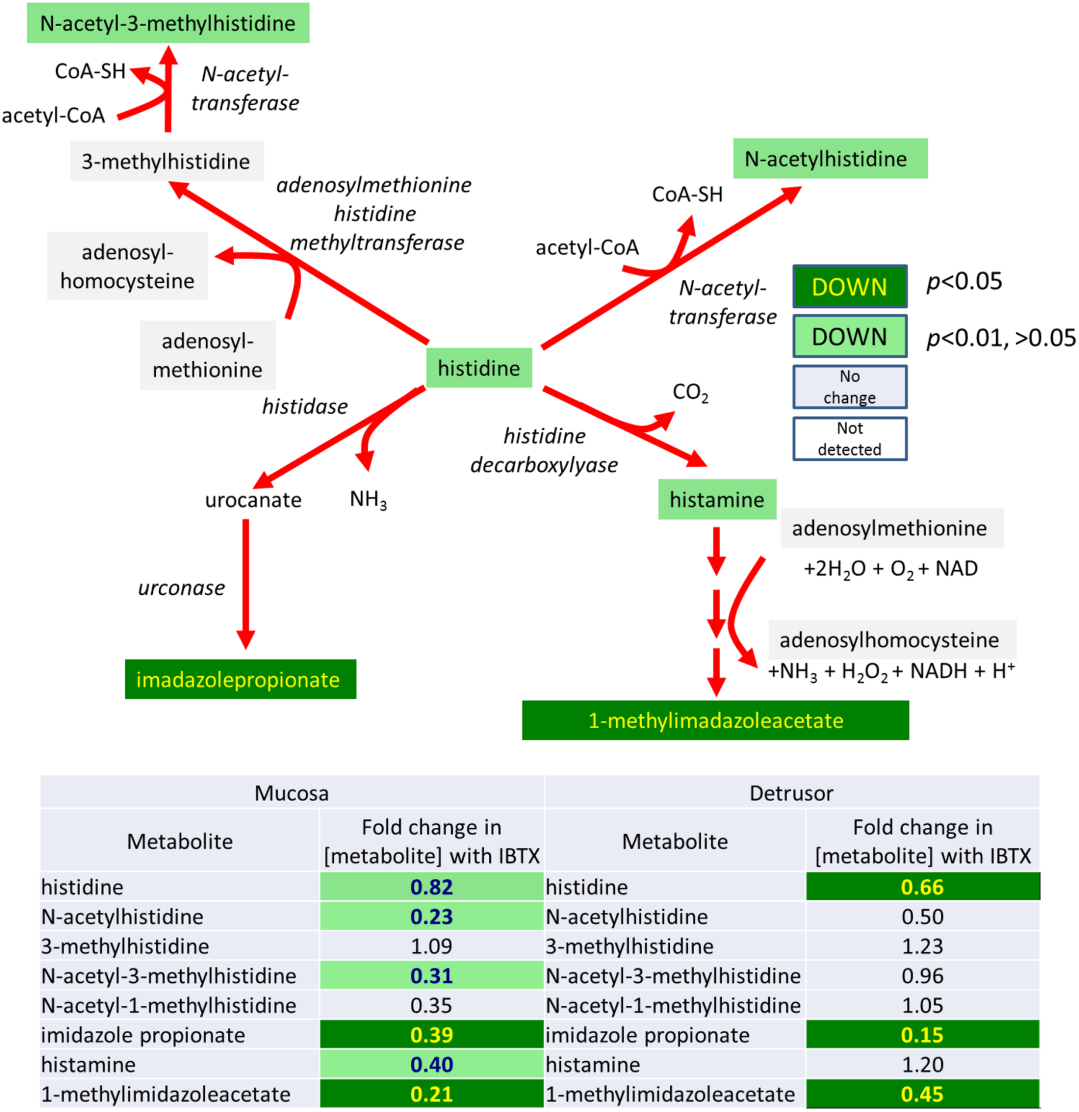
Iberiotoxin treatment causes a significant decrease in the histidine metabolism products in mucosal and detrusor tissue.

**Fig 11:**
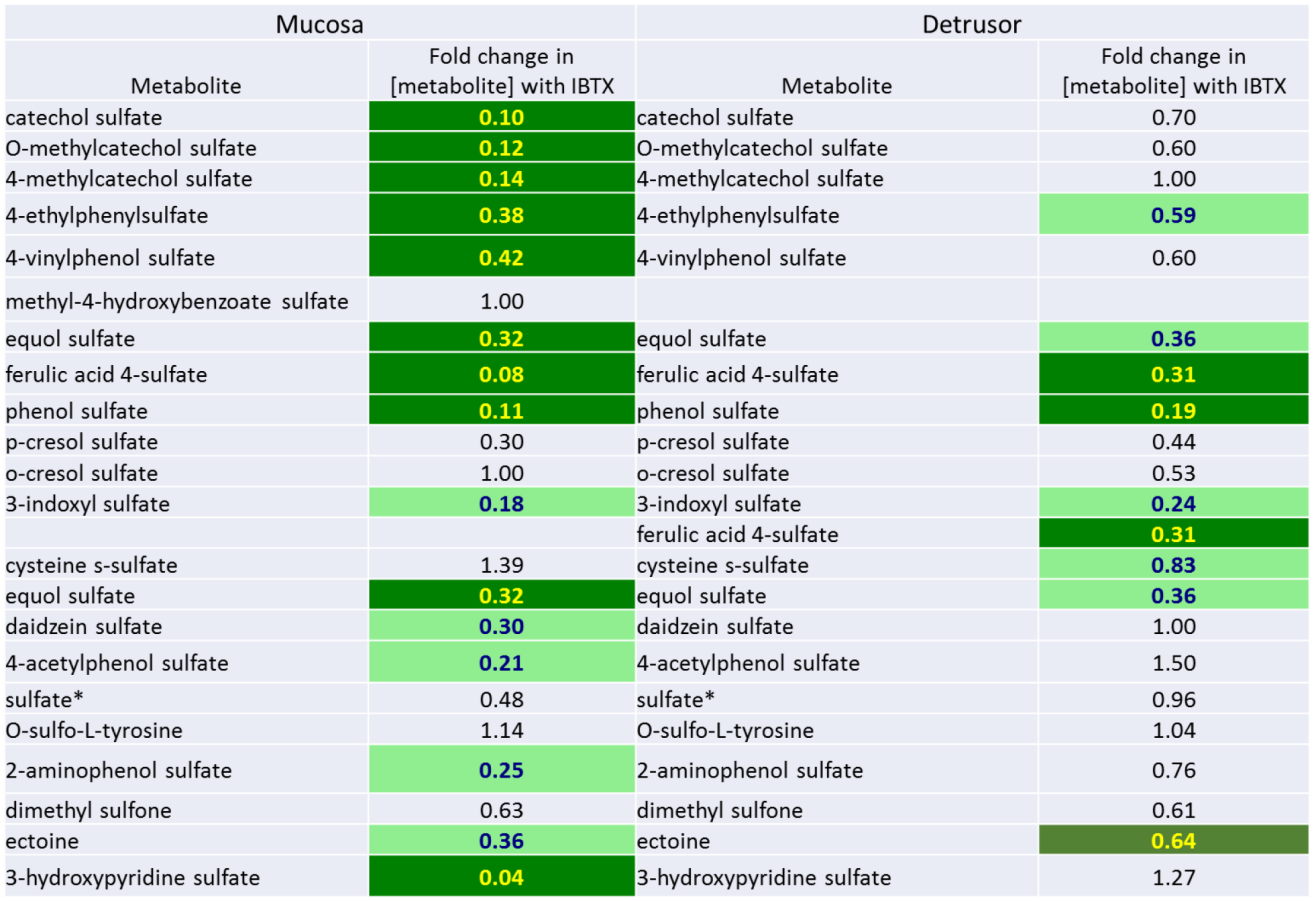
Comparative changes in levels of several sulfonated metabolites in the mucosa and detrusor following iberiotoxin treatment.

In addition to effects on metabolism that can be directly ascribed to inhibition of MaxiK-activity of mitochondrially-associated pathways there were some effects on predominantly cytosolic pathways. For example, as shown in Fig 10, an effect on the histidine/histamine pathways, and as shown in Fig 11, on sulfonation. However, several of the steps in histidine metabolism requires mitochondrially-derived co-factors, such as acetyl-CoA and NAD/NADH. Similarly, sulfotransferases, which add a sulfuryl group to metabolites, utilize the co-factor 3-phosphoadenosyl-5 phosphosulfate which has a synthetic pathway involving several mitochondrially-mediated steps. Therefore, although these pathways and reactions occur in the cytosol their down-regulation may be a reflection of lowered levels of mitochondrially-derived co-factors as a result of IBTX treatment.

### In the detrusor urothelial inhibition of MaxiK has additional specific effects on protein catabolism and energy generating pathways

As described above, there is a change in the levels of some amino acids and their N-acetylated products in both mucosal and detrusor tissue following IBTX treatment. However, as shown in Fig 7 the levels for nearly all amino acids are reduced in the detrusor (irrespective of polarity, or if they are essential amino acids). The almost uniform lowered levels of amino acids caused by IBTX treatment in the detrusor strongly suggests that there lowered catabolism of proteins (which occurs in the mitochondria).

**Fig 12:**
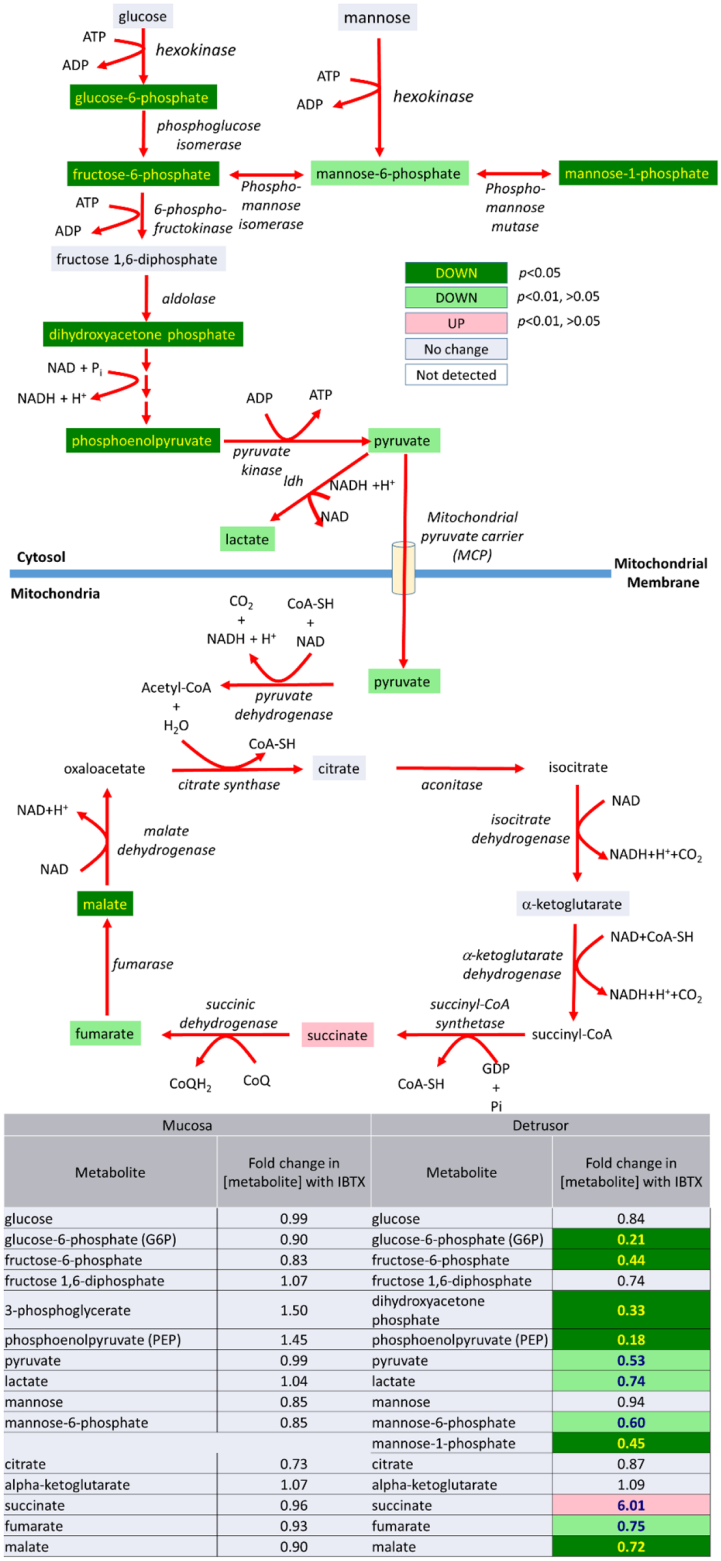
Iberiotoxin treatment causes a significant decrease in the energy generating pathways (glycolysis and the TCA cycle) in detrusor (but not mucosal) tissue.

In addition, there is an effect on the energy generating pathways of the detrusor, which is not seen in the mucosa (See Fig 12). In the detrusor levels of glucose and mannose levels are not affected, suggesting their cellular uptake, and derivation from glycogen stores, are unaffected by IBTX treatment. However there are decreased levels of several glycolytic intermediates. Although glycolysis occurs in the cytosol, several key enzymatic steps are dependent on co-factors produced in the mitochondria (eg. ATP and NADH). The TCA cycle is also perturbed at the level of succinate dehydrogenase (which is part of complex **I**II in the oxidative phosphorylation transport chain in the mitochondrial membrane). Overall, the change in energy pathways resulting from inhibition of urothelial MaxiK inhibition is only evident in the detrusor. These changes may be a result of IBTX effecting MaxiK-activity linked signaling pathways between the mucosa and detrusor which regulate detrusor energy generating pathways or may be secondary to changes in bladder physiology that affect the energy demands of the detrusor.

## Discussion

This report is the first to apply metabolomics to document global changes in bladder metabolism mediated through urothelial MaxiK-activity. Interestingly, IBTX inhibition of urothelial MaxiK-activity results in changes in metabolism in both the mucosa and detrusor. However, the changes in metabolism of mucosa and detrusor are significantly different, supporting previous work that different compartments of the bladder should be considered as separate entities [11]. Overall our work supports a growing body of evidence that the urothelium plays an important regulatory role in overall bladder function, not only in mediating solute transport, but also in sensing and communicating to other tissues, primarily the detrusor, to respond to bladder fullness [18]. A primary signaling mechanism to initiate this response is believed to be the action of ATP on P2X3 receptors of sensory nerves [19,20]. The observation that inhibiting urothelial MaxiK-activity inhibits mitochondrial energy metabolism would be expected to reduce overall cellular ATP levels and therefore potentially ATP mediated signaling. However, our studies do not distinguish between the levels of ATP in different cellular pools, for example ATP stored in cellular vesicles (that are proposed to be the source of ATP used in cellular signaling [21]) and pools used in energy generating/utilizing processes.

The conclusions in this paper are dependent on the specificity of IBTX to block vesicular MaxiK channels on the luminal side of the urothelial layer and thereby not affect underlying structures. Several publications have demonstrated the inability of IBTX to cross epithelial layers [1–3]. Although these publications show that IBTX does not cross epithelial layers, it has not been specifically shown for the bladder urothelium. However, since the urothelium forms the tightest and most impermeable barrier in the body [4] we are confident that IBTX will only affect MaxiK channels on the luminal side of the urothelium. In addition, the surgery to insert the infusion catheter into the bladder lumen, or over-distention of the bladder during infusion, may result in the loss of barrier integrity. However the surgery to insert the catheter was performed 4 days prior to the infusion of IBTX which would allow for healing of bladder tissue at the site of catheter entry. During the infusion of IBTX the bladder is not over-distended and several studies suggest that filling the bladder to capacity does not affect bladder permeability [5–7]. In addition, the surgery to insert the infusion catheter into the bladder lumen, or over-distention of the bladder during infusion, may result in the loss of barrier integrity. However the surgery to insert the catheter was performed 4 days prior to the infusion of IBTX which would allow for healing of bladder tissue at the site of catheter entry. During the infusion of IBTX the bladder is not over-distended and several studies suggest that filling the bladder to capacity does not affect bladder permeability [5–7]. Also, as with all studies using inhibitors, consideration needs to be given to the specificity of the inhibitor for its target. However, IBTX at the concentrations used in the present studies is considered a highly selectivity inhibitor of the MaxiK channel [22]. In GH3 cells IBTX does not alter A-type K^+^, Ca^2+^, or Na^+^ currents, but completely inhibits the MaxiK Ca^2+-^activated K^+^ currents present in this type of cell and in lympocytes, K^+^ channels are unaffected by IBTX [23,24]. Given its high specificity, IBTX is a commonly used and important tool to obtain information on the function of MaxiK channels.

Given that the effect of IBTX is primarily on the luminal side of the urothelial layer, the primary effect on mitochondrial metabolism is intriguing. A possible explanation is that as a result of inhibition of MaxiK channels there is increased membrane polarization, which in turn would activate voltage dependent calcium channels increasing cytosolic [Ca^2+^] levels, which are known to cause remodeling of mitochondria [25]. An alternative explanation is that IBTX disrupts the interaction of MaxiK with proteins, such as those involved in signal transduction (several potential candidates were recently identified as part of the MaxiK interactome) or translocation of MaxiK into the mitochondria (for example, through disrupting its interaction with Tom22) [26,27].

The effect of changes in metabolism in the mucosa are potentially coordinated through the spread of either [Ca^2+^] levels or signaling molecules through gap junctions (primarily Cx43) and pannexin 1 (Panx1). [28,29]. However, the effect on detrusor is most likely communicated via changes in signal molecule levels released from the mucosal tissue which either acts directly on the detrusor or via the nervous system. As described above the effect on mitochondrial metabolism would affect ATP, which is known to be involved in mucosal co-ordination of bladder function. However, the levels of several other metabolites effected by MaxiK inhibition could also potentially be involved with coordination of responses between the mucosa and detrusor. For example, histamine (down 2.5-fold in the mucosa), acetylcholine (down 50-fold in detrusor) and arachidonic acid derived metabolites which act as paracrine agents (prostacyclin, 12-HEPA and 12-HETE, both down approximately 2-fold in detrusor). In addition, the inhibition of mitochondrial energy metabolism would represent a switch from aerobic to anaerobic metabolism, in effect a “metabolic reprogramming” of cells, which has been shown to affect release of prostaglandins from endothelial cells [30,31].

There are several animal models that implicate bladder pathophysiology with changes in MaxiK activity. For examples, bladder from rabbits with partial urethral obstruction (PUO), a model of overactive bladder, protein levels of MaxiK are down-regulated [3]. IBTX increased contractile responses in urinary bladder smooth muscle (UBSM) strips isolated from diabetic compared to non-diabetic animals, but this was not associated with detectable changes in mRNA levels encoding the α- and β1-subunits of the MaxiK channel [32]. In contrast, a later report using patch-clamp electrophysiology demonstrated a reduction of MaxiK activity in UBSM cells isolated from diabetic compared to non-diabetic bladder, accompanied with increased α- and decreased β1-subunit mRNA expression [33]. A third study demonstrated that IBTX induced phasic activity in UBSM strips, an effect that was significantly enhanced in UBSM strips isolated from diabetic animals [34]. The apparent contradictions in these studies, that even though there is decreased MaxiK activity in the diabetic bladder [33] IBTX has greater effect on contractility of diabetic UBSM [32,34] could potentially be explained by early studies not considering the regulatory role that the urothelium plays in mediating bladder contractility. The effect of diabetes on MaxiK channel activity was only measured in detrusor cells whereas contractility studies on UBSM strips were not denuded of urothelium. Thus, if diabetes has differential effects on MaxiK activity in urothelium and detrusor, then diabetes-induced changes in the effect of IBTX on UBSM contractility maybe primarily mediated through the urothelium. Indeed, we and others [5,35] have published that the presence of the urothelium on UBSM strips from non-diabetic animals increases sensitivity of carbachol-induced contractility to IBTX, although this effect is attenuated with diabetes. The data presented here further establishes an important role for the urothelium in regulating bladder physiology, urothelial MaxiK activity not only regulates mucosal, but also detrusor activity.

Supporting an important role of urothelial MaxiK in regulating bladder physiology are our own studies in the PUO animal model have demonstrated that instillation of pVAX-Slo (a plasmid construct expressing MaxiK) into the bladder lumen reduced over-activity [4]. Given the conditions (luminal instillation) for gene transfer in these experiments it is unlikely that expression of MaxiK was increased in more than the most superficial tissues of the bladder (the urothelium) and yet impacted the whole physiology of the bladder.

Even within tissue compartments considered as part of our study (the mucosa and detrusor, which are the minimal units of the bladder which are easily separated with sufficient tissue to perform the metabolomics analysis) there are multiple cell types forming sub-compartments, such as interstitial (and nerve cells [36,37]. Therefore the metabolites measured must be considered as an average across all the present cell types in that tissue.

Although it is generally accepted that MaxiK channels are fundamental regulators of bladder physiology because of their involvement in detrusor excitability and contractility our work adds to the growing body of evidence that MaxiK activity in the urothelium may have a preeminent role in the regulation of bladder physiology. We demonstrate that urothelial MaxiK channel activity plays a significant role in determining mitochondrially-associated metabolism in mucosal tissues, which in turn can affect the metabolism of detrusor tissue. In the detrusor, changes in metabolism maybe a consequence of changes in the levels of mucosal signaling pathways, or secondary to changes in detrusor physiology, or a combination of both. Since decreased MaxiK activity is associated with several bladder pathophysiologies, the changes in mucosal metabolism reported here may represent new targets for pharmacological or genetic control of urinary bladder function in humans [38].

## Supporting information

S1 Fig All metabolites detected in bladder mucosa and detrusor, with the ratio of changed levels after iberiotoxin treatment. Significant changes following IBT treatment are indicated as follows; Dark Green Background: Indicates significant (p≤0.05) down-regulation of metabolite following IBTX treatment; Light Green Background: Indicates a trend/narrowly missed statistical significance (0.05<p<0.10) for down-regulation of metabolite following IBTX treatment; Dark Red Background: Indicates significant (p≤0.05) up-regulation of metabolite following IBTX treatment; Light Red Background: Indicates a trend/narrowly missed statistical significance (0.05<p<0.10) for up-regulation of metabolite following IBTX treatment. Grey Background: Indicates a detected metabolite which was unchanged following IBTX treatment. White Background: Indicates an undetected metabolite which is part of the metabolic pathway.
(TIF)
