# Supplementary figures and images for: Urothelial MaxiK-activity regulates mucosal and detrusor metabolism
Source: PLoS One. 2017 Dec 27;12(12):e0189387. doi: 10.1371/journal.pone.0189387 (PMC5744919; doi:10.1371/journal.pone.0189387)

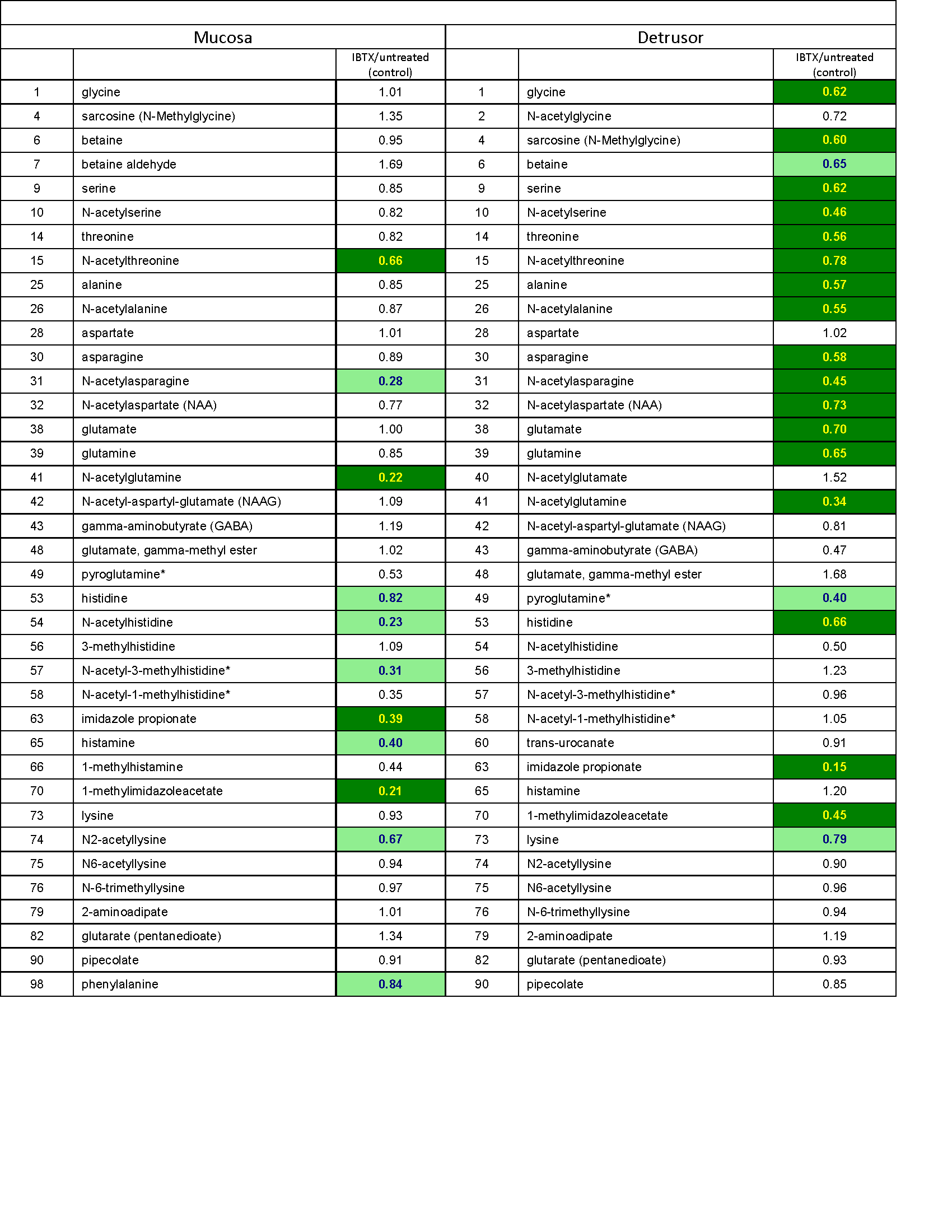

Supplement: S1 Fig — Significant changes following IBT treatment are indicated as follows; Dark Green Background: Indicates significant (p≤0.05) down-regulation of metabolite following IBTX treatment; Light Green Background: Indicates a trend/narrowly missed statistical significance (0.05<p<0.10) for down-regulation of metabolite following IBTX treatment; Dark Red Background: Indicates significant (p≤0.05) up-regulation of metabolite following IBTX treatment; Light Red Background: Indicates a trend/narrowly missed statistical significance (0.05<p<0.10) for up-regulation of metabolite following IBTX treatment. Grey Background: Indicates a detected metabolite which was unchanged following IBTX treatment. White Background: Indicates an undetected metabolite which is part of the metabolic pathway. (TIF) [file pone.0189387.s001.tif]
